# Impact on quality of life with affirmative feedback on weight loss after gastrectomy

**DOI:** 10.1038/s41598-023-42695-w

**Published:** 2023-09-18

**Authors:** Seung Soo Lee, Ho Young Chung, Oh Kyoung Kwon

**Affiliations:** 1 Department of Surgery, Kyungpook National University Hospital, School of Medicine, Kyungpook National University, 130 Dongdeok-ro, Jung-gu, Daegu, 700-721 Republic of Korea; 2 Department of Surgery, Kyungpook National University Chilgok Hospital, School of Medicine, Kyungpook National University, Daegu, Republic of Korea

**Keywords:** Gastroenterology, Health care, Medical research, Signs and symptoms

## Abstract

This study investigated the feasibility of improving post-gastrectomy satisfaction/quality of life (QoL) of gastric cancer survivors by readjusting their expectations through patient interviews. Weight loss after gastric cancer surgery is common, and a change in the departmental policy helped in providing cancer survivors with an alternative interpretation of lost weight. Under the new policy, a group of patients who were preoperatively overweight or obese were provided with affirmative feedback, despite their postoperative weight loss. The European Organization for Research and Treatment of Cancer QoL Questionnaire-C30 and -STO22 were used to assess the QoL. The postoperative 1-year QoL was compared before (control) and after policy changes (affirmative-feedback group) in preoperatively overweight (or obese) patients who lost weight. Despite the weight loss, the affirmative-feedback group exhibited a higher percentage of “less worried” responses (90.4%) on low body weight concerns as compared to the control group (76.5%; P = 0.037). Significant QoL advantages were also observed in the affirmative-feedback group on multiple scales (global health status/QoL, nausea/vomiting, diarrhea, dysphagia, dry mouth, and body image). Patient interviews with affirmative feedback on weight loss improved weight satisfaction and QoL in gastric cancer survivors who lost weight.

## Introduction

Gastric cancer survivors experience changes in quality of life (QoL) after surgery^1–4^, which include QoL deterioration of symptomatic and behavioral aspects in the postoperative period. While QoL deterioration in gastric cancer survivors occurs due to various symptoms, behavior related QoL deterioration is known to persist even longer^5^.

Clinicians are very concerned about patients’ QoL, including its management, which should be a greater part in cancer survivorship care. Depending on the onset of clinical effort, QoL management can be considered preemptive or reactive. While there are several studies on QoL management of patients with cancer, most of these focus on preemptive measures to minimize the occurrence of QoL deterioration by suggesting specific procedures or chemotherapeutic regimens with better QoL outcomes^6–11^. Reactive strategies for pre-existent QoL deterioration have only been presented as theories and hypotheses or outcomes of small-sized observational studies. These strategies include symptomatic medications, nutritional support, and patient interviews^1,5,12,13^.

By definition, QoL represents an individual’s overall satisfaction with life and well-being^14^. Their perceived position in their own set of values decides the QoL outcomes^15^. Thus, a reduction in the gap between the patient’s expectations and achievements is vital to QoL management^16^. Among the strategies suggested for the postoperative management of QoL deterioration, symptomatic medications and nutritional support aim at restoring specific objective outcomes or achievements, thus facilitating patient satisfaction. However, patient interviews aim at intervening in the patient’s value systems or the level of expectations; thus, moderating the mismatch between the ideal and reality. The versatility of patient interviews could become phenomenal because, at least in theory, controlling of the ideal (or level of expectation) can be applied to nearly any aspect of the clinical outcomes by modulating the gap between the patient’s expectations and achievements. However, despite these theoretical implications, the actual value of patient interviews on patient satisfaction and QoL outcomes has not been demonstrated yet in clinical settings.

Therefore, this study aimed to investigate the feasibility of modulating patient satisfaction/QoL by readjusting patient expectation levels through patient interviews and identify the role of patient interviews concerning cancer survivorship care. To achieve this, the QoL of gastric cancer survivors with weight loss were monitored after a series of interviews with affirmative feedback on weight loss.

## Methods

### Study groups and design

A considerable number of gastric cancer survivors were reported to undergo postoperative weight loss^17–19^. In 2015, a departmental policy to provide gastric cancer survivors with an interpretation of their current weight was set, based on the body mass index (BMI) chart recommended by the WHO Western Pacific Region for Asian adults at postoperative 3-, 6-, 9-, and 12-month follow-ups^20^ to allow survivors to understand their current weight status.

Through this policy, a group of patients who lost weight had a positive interpretation of their current weight despite the weight loss. While a typical weight comparison against the preoperative weight should yield a negative interpretation of one’s current weight for all those who lost weight, the new policy allowed those who were preoperatively overweight or with obesity to receive affirmative feedback about their weight loss.

This study assessed perceptional changes regarding lost weight as of 2015 among those who were overweight preoperatively or those with obesity and had lost weight at the postoperative 1-year follow-up. A wash-out period from January 1, 2014, to December 31, 2015 was set. The patients were divided into two groups: a control group comprising patients who underwent surgery between 2010 and 2013 and the affirmative-feedback group which included those who underwent surgery between 2016 and 2019.

**Figure 1:**
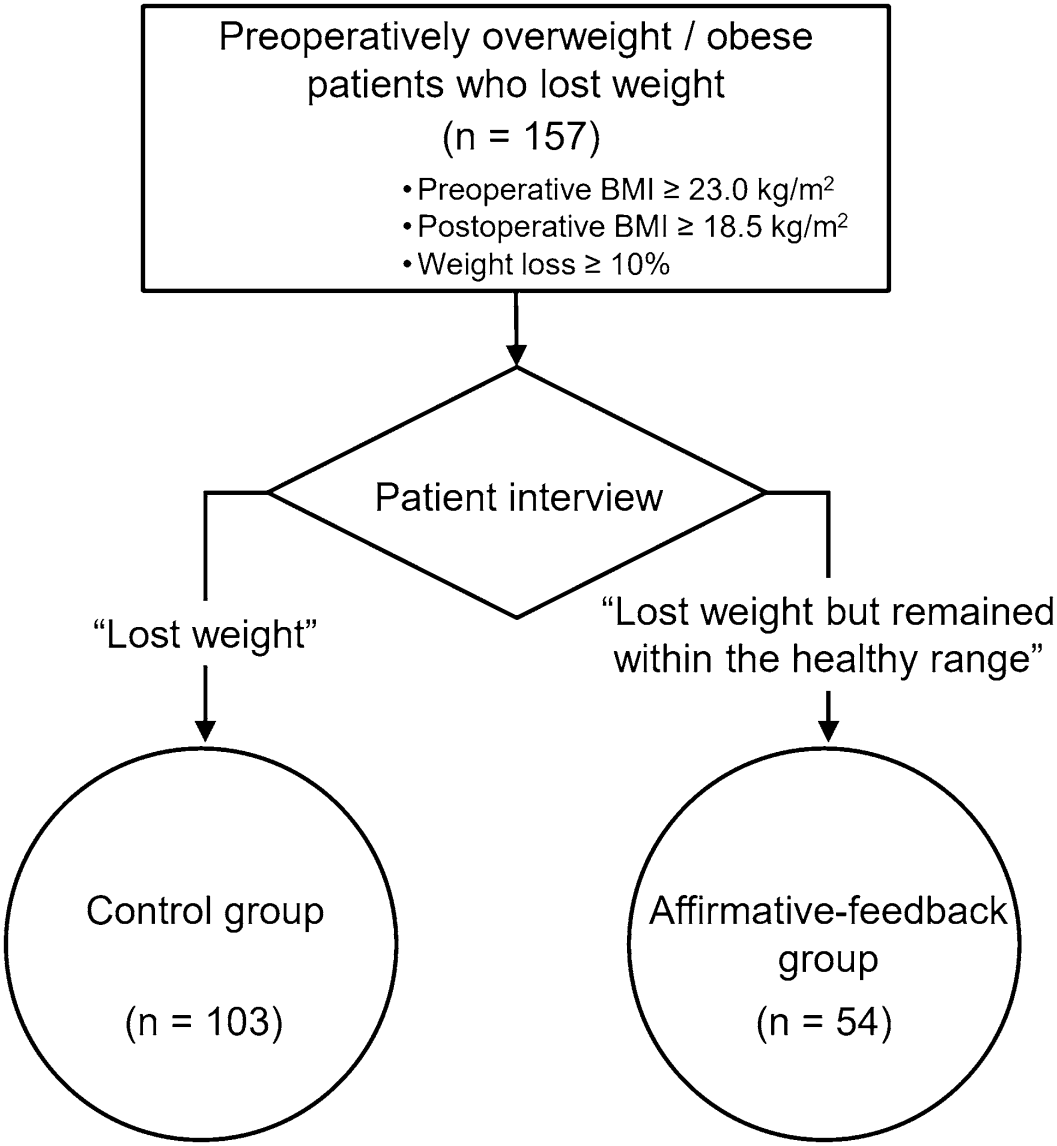
Overview of the study design, showing perceptional differences toward weight loss patients with gastric cancer who were overweight or with obesity based on the BMI chart recommended by the WHO Western Pacific Region for Asian adults. *BMI* body mass index.

Gastric cancer survivors who underwent curative resection for a primary gastric adenocarcinoma and later diagnosed with stage 1 disease according to the 8th edition of the Union for International Cancer Control (UICC) classification were considered eligible for the study. None of the patients underwent neoadjuvant chemotherapy. Those with higher stage cancer were not included, because advanced cancer status and consequent additional treatment efforts such as adjuvant chemotherapy might have a sustained impact on patients’ QoL and body weight^21,22^. Preoperatively overweight or patients with obesity (BMI ≥ 23.0 kg/m^2^) experiencing significant weight loss, with a total loss of ≥ 10% of their preoperative weight at the postoperative 1-year follow-up, were identified (Fig. 1). Those with excessive weight loss, which placed them in the underweight category (BMI < 18.5 kg/m^2^), were considered ineligible.

The postoperative 1-year QoL for 159 patients was assessed using the European Organization for Research and Treatment of Cancer (EORTC) QoL questionnaire (QLQ)-C30 and -STO22 from among the 190 patients who met the inclusion criteria of this study. However, two patients who were undergoing treatment for other malignancies were excluded. Finally, a total of 157 patients (103 and 54 patients in the control and affirmative-feedback groups, respectively) were included in this study.

### Ethical approval statement

This study was approved by the Institutional Review Board (IRB) of Kyungpook National University Hospital (No. KNUH 2021-07-062) and was conducted in accordance with the Declaration of Helsinki. Written informed consent was waived due to the retrospective nature of the study.

### Surgical and clinical protocol

The practice for gastric cancer has been standardized over the decades^23^. The principal surgical approach with curative intent for gastric cancer involves a gastrectomy and D2 lymph node dissection. The extent of gastrectomy is based on the location of the cancer. Distal subtotal gastrectomy is recommended for those with gastric cancer in the lower part of the stomach, while total gastrectomy for those with gastric cancer in the upper part of the stomach. Recommended reconstruction methods following a distal subtotal gastrectomy include Billroth I gastroduodenostomy, Billroth II gastrojejunostomy, and Roux-en-Y anastomosis, which is then finalized by the surgeon’s preference. Roux-en-Y anastomosis is suggested after a total gastrectomy. Moreover, both laparoscopic and open surgeries are proposed for stage 1 gastric cancer and postoperative adjuvant chemotherapy for those with gastric cancer of stage 2 or higher.

All the patients underwent gastrectomy and D2 lymph node dissection for stage 1 gastric cancer. Those with distal subtotal gastrectomy underwent either Billroth I gastroduodenostomy or Billroth II gastrojejunostomy, and those with total gastrectomy underwent Roux-en-Y anastomosis. Since all the included patients had stage 1 gastric cancer, a choice between laparoscopic and open surgeries was provided before the surgery. Patients resumed diet (starting off with sips of water) on the third postoperative day and were discharged on the sixth or seventh postoperative day. Since this study only included those with stage 1 cancer, none of them underwent chemotherapy.

### Affirmative feedback

The feedback consisted of a one-on-one session between the patient and attending physician, which lasted between 5 and 10 min for each session, with at least four scheduled sessions postoperatively (3-, 6-, 9-, and 12-month follow-ups) that were conducted in the outpatient department. When an affirmative feedback was applicable despite weight loss, they were provided with comments that their weight was within the healthy range. They were informed that returning to their preoperative weight could also mean gaining weight beyond the healthy range. The session continued until the patients stopped resisting the notion presented to them. The feedback was not limited to the scheduled sessions. Whenever this topic was brought up at another time during their visits to outpatient department, the same information was provided.

### QoL assessment

The Korean versions of EORTC QLQ-C30 and -STO22 were used to assess QoL^24^. Patients were asked to respond to 52 items by themselves, which were later converted into 24-scale scores of 0–100 according to the scoring manual. No response to any item was permitted, as originally designed by the EORTC.

Among the 52 items, item 48 pertained to personal concern about weight loss by asking the following question: “Did you worry about your weight being too low?” While responses to item 48 and two additional items were to be converted into a 0–100 score for the anxiety scale of the EORTC questionnaires, item 48 was analyzed to verify the individual’s perception of the lost body weight. The response of “not at all” or “a little” was categorized as “less worried,” and that of “quite a bit” or “very much” as “more worried.”

### Statistical analysis

For comparison of demographic data, chi-square tests were used to compare categorical variables, while Student’s t-tests to compare continuous variables. QoL changes at the postoperative 1-year follow-up were compared between the control and affirmative-feedback groups using a Student’s t-test. In particular, patient responses to item 48 were compared using a chi-square test. Continuous variables were presented in means and standard deviation. All the statistical analyses were performed using IBM SPSS Statistics for Windows, version 25.0 (IBM Corp., Armonk, NY, USA). A P-value of < 0.05 was considered statistically significant.

## Results

### Patient characteristics

There were no significant differences in sex and age between the control and affirmative-feedback groups (Table 1). The BMI of the control and affirmative-feedback groups were 25.7 ± 2.2 and 25.9 ± 2.1 preoperatively and 21.9 ± 1.9 and 21.8 ± 1.9 postoperatively, respectively, and there were no significant differences between the groups. There were no significant differences in the extent of postoperative weight loss between the control (10.1 ± 2.9) and affirmative-feedback (10.7 ± 3.5) groups.

**Table 1:** Characteristics of the study participants.

	Control group (n = 103)	Affirmative-feedback group (n = 54)	P-value
Sex			
Female	45 (43.7)	28 (51.9)	0.330
Male	58 (56.3)	26 (48.1)	
Age	60.3 ± 12.7	63.7 ± 9.7	0.066
Preoperative BMI (kg/m^2^)	25.7 ± 2.2	25.9 ± 2.1	0.714
Postoperative BMI (kg/m^2^)	21.9 ± 1.9	21.8 ± 1.9	0.802
Weight loss (kg)	10.1 ± 2.9	10.7 ± 3.5	0.308
Stage^†^			
Ia	86 (83.5)	48 (88.9)	0.364
Ib	17 (16.5)	6 (11.1)	
Resection			
Subtotal	73 (70.9)	38 (70.4)	0.948
Total	30 (29.1)	16 (29.6)	
Route			
Open	95 (92.2)	38 (70.4)	< 0.001*
Laparoscope	8 (7.8)	16 (29.6)	
Co-morbidity			
No	96 (93.2)	41 (75.9)	0.002*
Yes	7 (6.8)	13 (24.1)	
Cardiac	1	7	
Cerebrovascular	0	4	
Pulmonary	2	1	
Psychologic	1	1	
Hepatic	1	0	
Renal	1	0	
Immunologic	1	0	

Values are presented as n (%) or mean ± standard deviation.

*SD* standard deviation, *BMI* body mass index.

*Statistically significant difference between groups.

^†^Stage grouping according to the 8th edition of the Union for International Cancer Control classification.

Furthermore, regarding the minimally invasive surgery and aggressive treatment for patients at risk, the affirmative-feedback group (originating from the late 2010s) had more patients who underwent laparoscopic surgery (P < 0.001) and those with comorbidities (P = 0.002) than the control group (from the early 2010s). The percentage of Billroth I gastroduodenostomy following a distal subtotal gastrectomy was higher in the control (95.9%) and affirmative-feedback (73.7%) groups, showing that Billroth II gastrojejunostomy with laparoscope-friendly linear stapler was more preferred by surgeons.

### Personal concern about weight loss and QoL

When asked about personal concern about low body weight (item 48), the affirmative-feedback group exhibited a higher percentage of “less worried” responses (90.4%) than that in the control group (76.5%; P = 0.037; Table 2).

**Table 2:** Patient response to item 48 of the EORTC QLQ-C30 as compared between the control and affirmative-feedback groups at 1-year postoperatively.

	Control group	Affirmative-feedback group	P-value
Have you worried about your weight being too low?			
Not at all/a little	78 (76.5)	47 (90.4)	0.037*
Quite a bit/very much	24 (23.5)	5 (9.6)	

Values are presented as n (%).

*EORTC QLQ* European organization for research and treatment of cancer quality of life questionnaire.

*Statistically significant difference between groups.

The mean changes in the global health status/QoL for the control and affirmative-feedback groups were 2.2 ± 25.3 and 15.4 ± 27.8, respectively, in favor of the affirmative-feedback group (P = 0.008; Table 3) during a follow-up 1 year postoperatively. There were no significant differences in QoL changes according to the EORTC QLQ-C30 functional scales. However, the affirmative-feedback group exhibited QoL advantages according to the EORTC QLQ-C30 nausea/vomiting (P = 0.012) and diarrhea (P = 0.028) scales as well as EORTC QLQ-STO22 dysphagia (P = 0.007), dry mouth (P = 0.001), and body image (P = 0. 005) scales.

**Table 3:** QoL changes in the control and affirmative-feedback groups at 1-year postoperatively.

	Control group		Affirmative-feedback group		P-value
	R	Mean (SD)	R	Mean (SD)	
EORTC QLQ-C30					
Global health status/QoL^†^	94	2.2 (25.3)	41	15.4 (27.8)	0.008*
Functional scale^†^					
Physical functioning	103	− 4.1 (19.1)	54	− 2.2 (15.5)	0.523
Role functioning	103	− 10.7 (24.7)	54	− 4.0 (20.5)	0.091
Emotional functioning	103	1.2 (20.4)	54	4.4 (20.5)	0.355
Cognitive functioning	103	− 4.4 (18.2)	54	− 4.0 (18.0)	0.907
Social functioning	102	− 2.9 (30.6)	52	3.5 (21.0)	0.174
Symptom scales/items^‡^					
Fatigue	102	12.0 (23.9)	54	5.9 (20.8)	0.114
Nausea and vomiting	103	6.6 (23.5)	54	− 3.1 (21.0)	0.012*
Pain	103	3.6 (23.4)	54	1.9 (23.7)	0.666
Dyspnea	103	3.6 (26.0)	53	− 2.5 (26.0)	0.168
Insomnia	102	− 2.6 (28.4)	53	− 3.1 (25.5)	0.909
Appetite loss	103	8.1 (32.8)	54	1.9 (29.3)	0.243
Constipation	101	1.3 (25.8)	54	0.0 (28.2)	0.769
Diarrhea	99	15.5 (31.0)	54	4.9 (26.2)	0.028*
Financial difficulties	102	1.0 (34.9)	50	− 6.0 (25.8)	0.212
EORTC QLQ-STO22^‡^					
Dysphagia	103	8.4 (14.0)	53	1.8 (14.8)	0.007*
Pain	103	3.2 (17.9)	53	− 0.4 (17.0)	0.225
Reflux	103	4.6 (22.5)	53	− 2.4 (19.5)	0.055
Eating restrictions	103	10.6 (17.6)	53	7.7 (16.3)	0.312
Anxiety	103	11.1 (22.3)	53	3.9 (22.4)	0.057
Dry mouth	102	6.5 (26.6)	52	− 9.0 (28.1)	0.001*
Taste	102	8.8 (27.7)	52	1.9 (22.3)	0.098
Body image	100	19.0 (37.1)	52	4.5 (25.6)	0.005*
Hair loss	18	− 5.6 (46.1)	9	7.4 (36.4)	0.470

*QoL* quality of life, *R* number of responders, *SD* standard deviation, *EORTC QLQ* European organization for research and treatment of cancer quality of life questionnaire.

*Statistically significant difference between groups.

^†^Positive values indicate QoL improvement.

^‡^Positive values indicate QoL deterioration.

## Discussion

This study investigated personal satisfaction and QoL associated with postoperative weight loss among preoperatively overweight or obese gastric cancer survivors with/without affirmative feedback. Despite a comparable extent of weight loss due to surgery, those with affirmative feedback were more satisfied with their current weight and QoL.

QoL represents patients’ perception of their position in life in the context of their culture and value system^15^. There are reports indicating the possibility of QoL manipulation by patient-perception interventions. In particular, patients who undergo laparoscopic surgery may experience a reduction in early QoL if they had high expectations of the laparoscopic surgery, neglecting the equivalent extent of gastrectomy by both open and laparoscopic surgeries^25^. Another study showed comparable QoL between patients with actual early-stage cancer and those with presumed early- but late-stage cancer during the preoperative period^21^. This indicates that processing of clinical information may be the potential target of QoL management.

In theory, vegetarians or non-drinkers enjoy social meals once they have their value system on social meals readjusted from the consumption of alcohol or meat to social interaction with others^26,27^. This may also apply to patients who have undergone gastrectomy and have a limited food reservoir^5^. Even if they cannot consume foods in the same way as others, they should be able to enjoy social meals if they focus on their interaction with others. Thus, intervening in patients’ value systems and their interpretation of their current status has the potential of adjusting and improving QoL.

Efforts to intervene in the patient’s values or perception should not be justified without a reasonable or ethical basis. The modified post-gastrectomy surveillance protocol in this study, which included weight interpretation based on the well-accepted BMI chart recommended by the WHO Western Pacific Region for Asian adults, allowed for the adjustment of processing of clinical information i.e., the postoperative weight. Under the modified protocol used in this study, a group of gastric cancer survivors who were overweight or obese was provided with an interpretation of their current weight as “decreased, but healthier,” and the patients’ satisfaction regarding their weight and QoL were found to be significant 1 year after surgery.

A previous study showed that patients with gastric cancer who were obese had spontaneous satisfaction with weight loss 5 years after the surgery^28^. In that study, no clinical efforts were made to alter the information processing of the patients regarding their weight loss, and this self-justification was not yet apparent at 1-year follow-up postoperatively. The effort in this study in providing affirmative feedback about weight loss seems to have significantly reduced the time needed for the patients to accept their weight and showed increased satisfaction and better QoL even at 1-year follow-up postoperatively. Modern medicine is highly focused on improving patients’ QoL and clinicians are trying to provide patients with as many benefits regarding QoL as possible, even if for a brief period. For example, the QoL benefits of laparoscopic gastric cancer surgery over open surgery are known to be limited to a brief early postoperative period; nevertheless, time and effort is expended in laparoscopic surgery to provide these benefits. The study revealed the QoL benefits of affirmative feedback for a substantial period, suggesting it to be effective as well as efficient in QoL management of cancer survivors. Thus, affirmative feedback deserves high priority in cancer survivorship care.

Furthermore, patients provided with affirmative feedback exhibited better weight satisfaction as well as improved QoL. To understand QoL differences between groups, the focus needs to be placed on the probable behavior of those with weight dissatisfaction. Dissatisfied individuals would eventually attempt to gain weight by increasing dietary intake^29^, and such an effort goes against the dietary recommendations for patients who have undergone gastrectomy^30,31^. Consequently, they will be exposed to several gastrointestinal symptoms, such as nausea, vomiting, diarrhea, and dysphagia, which would deteriorate the overall QoL.

Survivors provided with affirmative feedback about weight loss showed improved QoL in terms of symptoms as well as in their perception of their body image according to EORTC QLQ-STO22. The body image scale is calculated from patient responses to the question “Have you felt physically less attractive as a result of your disease or treatment?” Despite the fact that both groups had lost weight, those provided with affirmative feedback had a more favorable interpretation of their weight loss (WHO BMI chart) and exhibited a more attractive outlook. Providing patients with a fair comparison seems to be an important factor in maintaining a positive outlook on one’s self-image after surgery.

The analysis of patient responses to item 48 showed more of less-worried responses about weight loss by the affirmative-feedback group. However, the adequacy of item 48 in representing patient satisfaction needs to be discussed. Items of the EORTC QLQs include two different questions, i.e., if the patient had certain symptoms (e.g., "Have you had pain?") and if certain symptoms upset them (e.g., "Did pain interfere with your daily activities?" or "Were you upset by the loss of your hair?"). While the former asks about the existence of a symptom, the latter focuses more on the personal interpretation or impact of the symptom. Therefore, the items in the latter question should be more capable of representing patient satisfaction regarding a certain symptom. Item 48 is similar to the latter question, which inquires if the low body weight worried the patient, and thus should be able to adequately reflect patients’ satisfaction regarding their current body weight.

Patient responses to items 47, 48, and 50 of the EORTC QLQ-STO22 were transformed into a 0–100 score on the anxiety scale. Even though the specific analysis of item 48 regarding weight loss concern revealed a significant advantage in the affirmative-feedback group, there was no significant difference in the anxiety score. Responses to items 47 (illness concern) and 50 (future health concern) may have mitigated the overall outcome.

The unique contribution of the current study is that it presents vital evidence showing that the level of satisfaction and QoL of patients with cancer can be modulated through patient interviews. Patients keep evaluating themselves based on the available value system. Upon giving the patients a favorable interpretation of their current status, patients will also reevaluate themselves favorably. This suggests a new scope of cancer survivorship care. In this study, cancer survivors had their target for weight comparison redirected from preoperative weight to that of the general healthy population. Future studies should focus on understanding the relevance of the alternative targets. If the alternative target could further be redirected beyond the state of the general population to that of average cancer survivors, a larger number of patients may have increased satisfaction with better QoL after cancer surgery. The difference in the nuance between “doing as good as others” and “doing as good as other cancer survivors” may lead to a huge difference in the magnitude of potential beneficiaries of the redirected target.

This study had some limitations. First, this study was a retrospective analysis before and after the surveillance protocol revision; therefore, it was influenced by time and changes in clinical trends. The number of patients who underwent laparoscopic surgery increased over time^32^, and the proportions of laparoscopic and open surgeries were significantly different between the groups. While improved QoL has frequently been reported, it is likely limited to the early postoperative period^9,33^. The study period was set at the postoperative 1-year follow-up; therefore, QoL influences of laparoscopic surgery would have likely been diminished by then. Second, there was a larger number of patients with comorbidities undergoing surgery over time. Protocols related to surgical decisions for patients with comorbidities were not changed, and such an increase would likely reflect the increased referral of patients with comorbidities for surgery in an aging society^34–36^. Finally, the sample size of the affirmative-feedback group was smaller as compared to that of the control group. Data from a larger pool would allow with in-depth analyses accounting for various patient factors.

## Conclusion

The study concluded that offering patient interviews with affirmative feedback about their weight loss facilitates greater weight satisfaction even in the first year following gastric cancer surgery. Moreover, becoming less keen on gaining weight, those with affirmative feedback enjoy better overall QoL, self-image, and digestive symptom scores. Therefore, efforts to ameliorate QoL deterioration in cancer survivors should be directed toward the provision of patient interviews/affirmative feedback following surgery.

## Data Availability

The datasets used and analyzed during the current study are available from the corresponding author upon reasonable request.
